# RNA-Mediated Gene Duplication and Retroposons: Retrogenes, LINEs, SINEs, and Sequence Specificity

**DOI:** 10.1155/2013/424726

**Published:** 2013-08-01

**Authors:** Kazuhiko Ohshima

**Affiliations:** Graduate School of Bioscience, Nagahama Institute of Bio-Science and Technology, Nagahama 526-0829, Japan

## Abstract

A substantial number of “retrogenes” that are derived from the mRNA of various intron-containing genes have been reported. A class of mammalian retroposons, long interspersed element-1 (LINE1, L1), has been shown to be involved in the reverse transcription of retrogenes (or processed pseudogenes) and non-autonomous short interspersed elements (SINEs). The 3′-end sequences of various SINEs originated from a corresponding LINE. As the 3′-untranslated regions of several LINEs are essential for retroposition, these LINEs presumably require “stringent” recognition of the 3′-end sequence of the RNA template. However, the 3′-ends of mammalian L1s do not exhibit any similarity to SINEs, except for the presence of 3′-poly(A) repeats. Since the 3′-poly(A) repeats of L1 and Alu SINE are critical for their retroposition, L1 probably recognizes the poly(A) repeats, thereby mobilizing not only Alu SINE but also cytosolic mRNA. Many flowering plants only harbor L1-clade LINEs and a significant number of SINEs with poly(A) repeats, but no homology to the LINEs. Moreover, processed pseudogenes have also been found in flowering plants. I propose that the ancestral L1-clade LINE in the common ancestor of green plants may have recognized a specific RNA template, with stringent recognition then becoming relaxed during the course of plant evolution.

## 1. RNA-Mediated Gene Duplication and Retroposons

### 1.1. Retrogenes and Processed Pseudogenes

**Figure 1:**
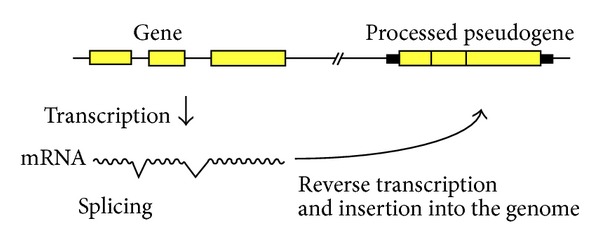
Schematic representation of the formation of a processed pseudogene.

Gene duplication is a fundamental process of gene evolution [1]. There are two types of gene duplication: direct duplication of genomic DNA and retropositional events [2–4]. Processed pseudogenes (PPs) are reverse-transcribed intronless cDNA copies of mRNA that have been reinserted into the genome (Figure 1) [5, 6]; they are especially abundant in mammalian genomes [7, 8]. PPs are not usually transcribed because they lack an external promoter; therefore, they have long been viewed as evolutionary dead ends with little biological relevance. However, recent studies have unveiled a substantial number of “processed genes” or “retrogenes” with novel functions that are derived from the mRNA of various intron-containing genes [9–12]. Molecular biological studies showed that a class of mammalian retroposons, long interspersed element-1 (LINE1, L1), has been involved in the reverse transcription of nonautonomous retroposons, such as PPs (retrogenes) and short interspersed elements (SINEs) [13].

### 1.2. Retroposons

**Figure 2:**
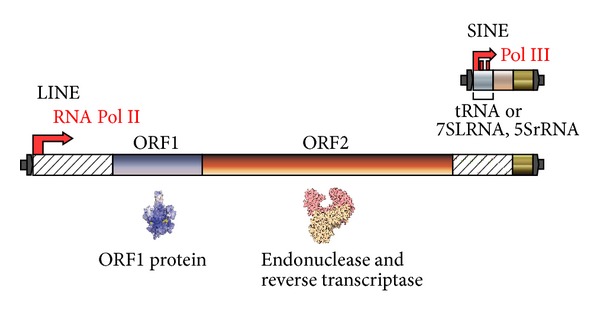
Schematic representation of a SINE and a LINE that have the same 3′-end sequence. Three-dimensional protein structures are taken from the L1-encoded ORF1 protein [94] and the reverse transcriptase of human immunodeficiency virus type 1 [95].

Eukaryotic genomes generally contain an extraordinary number of retroposons such as long terminal repeat (LTR) retrotransposons, LINEs or non-LTR retrotransposons, and SINEs [6, 14, 15]. LINEs have been characterized as autonomous retroposons bearing either one or two open reading frames (ORFs); all LINEs encode a reverse transcriptase (RT), and some, but not all, encode an apurinic/apyrimidinic endonuclease, a ribonuclease H, and/or putative nucleic-acid-binding motifs (Figure 2). Most members of a LINE family are truncated at various positions in their 5′ regions, constituting defective members of the family, the lengths of which range from 100 to 1,000 bp [13]. 

The Bombyx R2 LINE protein, which has sequence-specific endonucleolytic and RT activity, makes a specific nick in one of the DNA strands at the insertion site and uses the 3′ hydroxyl group that is exposed by this nick to prime the reverse transcription of its RNA transcript [16]. This mechanism is referred to as target DNA-primed reverse transcription (TPRT). The last 250 nucleotides that correspond to the 3′-untranslated region (UTR) of the R2 transcript are critical for this reaction [17]. Other LINEs, such as L1, are also believed to retrotranspose by TPRT [18]. The human L1 TPRT machinery has been reconstructed *in vitro* [19].

SINEs are non-autonomous retroposons, the 5′-end sequences of which are derived from tRNA, 5S rRNA, or 7SL RNA with promoter activity for RNA polymerase III (Figure 2) [20–22]. On the other hand, the 3′-end sequences of SINEs generally originated from a corresponding LINE [23]. A small nucleolar RNA-derived short retroposon, which lacks internal promoters for RNA polymerase III and has therefore not been subject to multiple rounds of retroposition, was recently discovered in the platypus [24]. 

### 1.3. Evolutionary Relationships of Various LINEs

Eickbush's group conducted comprehensive phylogenetic analysis of LINEs using extended sequence alignment of their RT domains [25]. All identified LINEs were grouped into 11 distinct clades. Assuming vertical descent, the phylogeny suggests that LINEs are as old as eukaryotes, with each of the 11 clades dating back approximately 2 billion years [25]. Currently, almost 30 clades have been recognized [26]. Mammalian L1s belong to the L1 clade, which includes numerous LINEs from vertebrates, slime mold, plants, and algae [25, 27, 28]. Analyses of L1-encoded endonucleases from zebrafish and mammals revealed that they are divided into 3 groups: M, F, and Tx1 [29]. Kordiš et al. showed that the genomes of deuterostomes possess three highly divergent groups of L1-clade LINEs, which are distinct from Tx group [28]. The Tx group, with a target-specific insertion, consists of 2 branches, one of which includes frog Tx1 [30].

### 1.4. SINEs and LINEs

**Figure 3:**
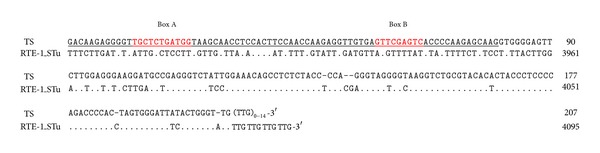
Sequence comparison of tobacco TS SINE with its partner LINE. The entire sequence of the TS SINE was aligned with the 3′-end sequence (~200 nucleotides) of a potato RTE-clade LINE. Dots and hyphens represent identical nucleotides and gaps, respectively. The tRNA-related region of the SINE is underlined, with the promoter sequences for RNA pol III (A & B boxes) highlighted in red. Nucleotide positions are shown on the right.

The 3′-end sequences of various SINEs originated from a corresponding LINE (Figure 2) [31]; for reviews, see also [23, 32, 33]. A systematic database and literature survey identified 58 SINEs, each possessing a common 3′-end sequence with its partner LINE (Table 1) [34]. For example, Figure 3 shows the alignment of tobacco TS SINE [35] with its partner LINE. This LINE, which was recently identified in the potato genome, a member of the same family as tobacco, belongs to the RTE clade. The 3′-end sequence of the SINE, approximately 100 bases, is nearly identical to that of the LINE, and they both end in TTG repeats [34]. SINE/LINE pairs have been observed in a wide variety of species, from eumetazoans to green plants, confirming the generality of this phenomenon (Table 1). Although various LINEs appear in the list, those from clades CR1 and RTE were particularly predominant.

Since the R2 LINE protein specifically recognizes the sequence near the 3′-end of the RNA transcript for the initiation of first-strand synthesis [16, 17], the homology between the 3′-ends of SINEs and LINEs suggests that each SINE family recruits the enzymatic machinery for retroposition from the corresponding LINE through this common “tail” sequence [31]. This hypothesis was strongly supported by experiments with SINE sequences in the eel [36]. As the 3′-UTRs of several LINEs have been shown to be essential for retroposition [17, 36–39], these LINEs presumably require “stringent” recognition of the 3′-end sequence of the RNA template [32, 36].

**Figure 4:**
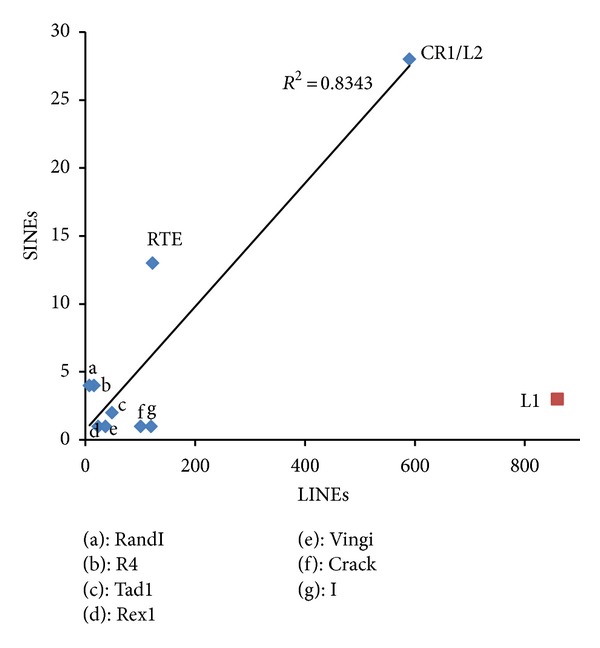
Relationship between the number of SINE/LINE pairs and the number of LINEs in each clade. The vertical axis shows the number of SINEs with a LINE tail [34]. The horizontal axis shows the number of LINEs belonging to each clade. The linear regression line, determined by the least squares approach, is shown, except for L1. *R* ^2^ indicates the coefficient of determination. CR1-clade LINEs (580 families) and L2-clade LINEs (10 families) were summed due to their confusing nomenclature.

Figure 4 illustrates the relationship between the number of SINE/LINE pairs and the number of LINEs in each clade [34]. Although Spearman's rank correlation is not significant (*ρ* = 0.25), the number of SINEs with a LINE tail is positively correlated with the number of LINEs belonging to each clade (*R* ^2^ = 0.83); that is, more LINEs tend to lead to more SINE/LINE pairs. Therefore, although a few LINE clades are the predominant source of SINE/LINE pairs, it is plausible that this simply reflects the large number of LINEs in these clades. However, L1-clade LINEs are the only prominent exception to this. Although over 800 L1-clade LINEs appeared in the database, only 3 SINEs with L1 tails were found [34], suggesting that, in general, L1-clade LINEs are different from other LINEs with regard to 3′-end recognition.

### 1.5. Mechanism of RNA-Mediated Gene Duplication in Mammals

Mammalian PPs and retrogenes were probably mobilized by L1s because they end in poly(A), and have L1-type target site duplications; they are inserted in L1-type endonuclease cleavage sites [40–42]. Molecular biological studies have shown that mammalian L1-encoded proteins have been involved in the reverse transcription of PPs [43, 44]. In the same assay, another class of autonomous retroposons, LTR retrotransposons (retroviral-like elements), were unable to produce similar PP-like structures [43].

The 3′-end sequences of mammalian L1 LINEs do not exhibit any similarity to SINEs, except for the presence of 3′-poly(A) repeats, although these L1s are thought to have mediated the retroposition of mammalian SINEs such as primate Alu and rodent B1 families [45–47]. Since the 3′-poly(A) repeats of L1 and Alu are critical for their retroposition in the HeLa cell line [46, 48, 49], L1 probably recognizes the 3′-poly(A) repeats. Therefore, while mammalian L1s do not require stringent recognition of the 3′-end sequence of the RNA templates, they are able to initiate reverse transcription in a more “relaxed” manner [32].

L1-encoded proteins are *cis*-acting; that is, L1 proteins preferentially mobilize or interact with the RNA molecule that encoded them [43, 44]. However, L1 is also thought to mobilize SINE RNAs and cytosolic mRNAs by recognizing the 3′-poly(A) tail of the template RNAs in *trans*, resulting in enormous SINE amplification and PP formation [43, 50]. Given that the L1 retropositional machinery acts in a *cis*-manner, Boeke [51] proposed the poly(A) connection hypothesis to explain why Alu RNA is mobilized by L1 at such a high frequency.

Schmitz et al. discovered a novel class of retroposons that lack poly(A) repeats in mammals. Termed tailless retropseudogenes, they are derived from truncated tRNAs and tRNA-related SINE RNAs [52]. To explain this phenomenon, they proposed a novel variant mechanism, probably guided by the L1 RT, in which neither the presence of a poly(A) tail on the RNA template nor its length is important for retroposition.

## 2. Retroposition Burst in Ancestral Primates

Abundant PPs are a feature of mammalian genomes [7, 8]. Previously, my collaborators and I performed the first comprehensive analysis of human PPs using all known human genes as queries [50]. We found the possibility of a nearly simultaneous burst of PP and Alu formation in the genomes of ancestral primates. The human genome was queried and 3,664 candidate PPs were identified; the most abundant of which were copies of genes encoding keratin 18, glyceraldehyde-3-phosphate dehydrogenase, and ribosomal protein L21. A simple method was developed to estimate the level of nucleotide substitutions (and therefore the age) of the PPs. A Poisson-like age distribution was obtained with a mean age close to that of the Alu repeats. These data suggested a nearly simultaneous burst of PP and Alu formation in the genomes of ancestral primates. Similar results have been reported by other groups [53–55]. The peak period of amplification of these 2 distinct retroposons was estimated to be 40–50 million years ago (mya) [50]; moreover, concordant amplification of certain L1 subfamilies with PPs and Alus was observed. We proposed a possible mechanism to explain these observations in which the proteins encoded by members of particular L1 subfamilies acquired an enhanced ability to recognize cytosolic RNAs *in trans*.

Roy-Engel's group recently recreated and evaluated the retroposition capabilities of two ancestral L1 elements, L1PA4 and L1PA8, which were active ~18 and ~40 mya, respectively [56]. Relative to the modern L1PA1 subfamily, they found that both elements were similarly active in a cell culture retroposition assay in the HeLa cell line, and both were able to efficiently *trans*-mobilize Alu elements from several subfamilies. They found limited evidence of differential associations between Alu and L1 subfamilies, suggesting that other factors are likely the primary mediators of their changing interactions over evolutionary time. Population dynamics and stochastic variation in the number of active source elements likely played an important role in individual LINE or SINE subfamily amplification [56]. If coevolution also contributed to changing retroposition rates and the progression of subfamilies, cell factors were likely to play an important mediating role in changing LINE-SINE interactions over evolutionary time.

We hypothesized that many human retrogenes were created during this period and that such retrogenes were involved in generating new characteristics specific to simian primates [50]. Several intriguing examples of primate retrogenes have been reported, for example, the human brain-specific isotype of the glutamate dehydrogenase (*GLUD2*) gene [57], the brain- and testis-specific *CDC14Bretro* gene, which evolved from the *CDC14B* cell cycle gene [58], and a novel chimeric retrogene (*PIPSL*) created by a unique mechanism [59–61], emerged by retroposition in a hominoid ancestor [54, 55, 62–65].

## 3. A Primate Retrogene That Was Created by a Novel Mechanism

### 3.1. Gene Creation by the Coupling of Gene Duplication and Domain Assembly

Most new genes arise by the duplication of existing gene structures, after which, relaxed selection on the new copy frequently leads to mutational inactivation of the duplicate; only rarely will a new gene with a modified function emerge. My collaborators and I described a unique mechanism of gene creation, whereby new combinations of functional domains are assembled at the RNA level from distinct genes, and the resulting chimera is then reverse-transcribed and integrated into the genome by the L1 retrotransposon [59]. We characterized a novel gene, which we termed *PIP5K1A* and *PSMD4-like* (*PIPSL*), created by this mechanism from an intergenic transcript between the phosphatidylinositol-4-phosphate 5-kinase (*PIP5K1A*) and the 26S proteasome subunit (*PSMD4*) genes in a hominoid ancestor. *PIPSL* is transcribed specifically in the testis of humans and chimpanzees and is posttranscriptionally repressed by independent mechanisms in these primate lineages. The *PIPSL* gene encodes a chimeric protein combining the lipid kinase domain of *PIP5K1A* and the ubiquitin-binding motifs of *PSMD4*. Strong positive selection on *PIPSL* led to its rapid divergence from the parental genes, forming a chimeric protein with distinct cellular localization and minimal lipid kinase activity, but significant affinity for cellular ubiquitinated proteins [59]. PIPSL is a tightly regulated, testis-specific novel ubiquitin-binding protein formed by an unusual exon-shuffling mechanism in hominoid primates and represents a key example of the rapid evolution of a testis-specific gene.

### 3.2. Evolutionary Fate of Primate *PIPSL*

Domain shuffling has provided extraordinarily diverse functions to proteins; nevertheless, how newly combined domains are coordinated to create novel functions remains a fundamental question of genetic and phenotypic evolution. My group presented the first evidence for the translation of PIPSL in humans [61]. The human *PIPSL* locus showed low nucleotide diversity within 11 populations (125 individuals) compared with other genomic regions, such as introns and overall chromosomes. It was equivalent to the average for the coding sequences or exons from other genes, suggesting that human *PIPSL* has some function and is conserved among modern populations. Two linked amino acid-altering single-nucleotide polymorphisms were found in the PIPSL kinase domain of non-African populations. They are positioned in the vicinity of the substrate-binding cavity of the parental PIP5K1A protein and change the charge of both residues. The relatively rapid expansion of this haplotype might indicate a selective advantage for it in modern humans [61]. 

**Figure 5:**
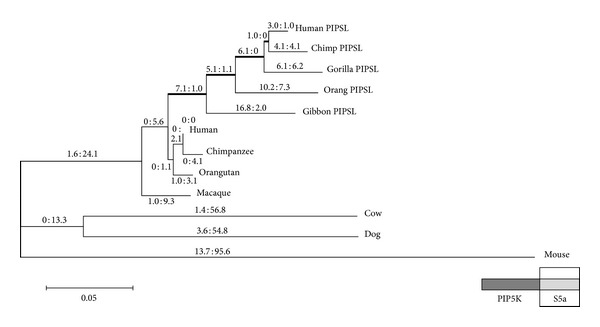
Molecular phylogeny and pattern of nucleotide substitutions of the *S5a*-derived region of *PIPSL* from all hominoid lineages [61]. The branches are drawn in proportion to the number of substitutions, with nonsynonymous (n) and synonymous (s) substitutions shown above each branch (n : s). An ancestral *PIPSL* lineage (indicated by bold lines) gradually accumulated 19 nonsynonymous and 2 synonymous substitutions. Since the split from the ancestral lineage, all the respective lineages have accumulated synonymous substitutions, except for gibbons, which still have a high n : s ratio (17 : 2).

We determined the evolutionary fate of PIPSL domains created by domain shuffling [61]. During hominoid diversification, the S5a/PSMD4-derived domain was retained in all lineages, whereas ubiquitin-interacting motif (UIM) 1 in the domain experienced critical amino acid replacements at an early stage, being conserved under subsequent high levels of nonsynonymous substitutions to UIM2 and other domains, suggesting that adaptive evolution diversified these functional compartments (Figure 5) [61]. Conversely, the PIP5K1A-derived domain is degenerated in gibbons and gorillas. These observations provide a possible scheme of domain shuffling in which the combined parental domains are not tightly linked in the novel chimeric protein, allowing for changes in their functional roles, leading to their fine-tuning. Selective pressure toward a novel function initially acted on one domain, whereas the other experienced a nearly neutral state. Over time, the latter also gained a new function or was degenerated. 

## 4. RNA-Mediated Gene Duplication in Land Plants

The SINE/LINE relationship in land plants is controversial. The first SINE/LINE pair of land plants was reported recently in maize [66]. However, the three tRNA-derived SINE families in *Arabidopsis thaliana* do not exhibit any similarity to the only LINE family (*ATLN*) in its genome [67–69]. Deragon's group proposed that the SINE-LINE relationship in *Arabidopsis* is not based on primary sequence identity but on the presence of a common poly(A) region [68]. 

I systematically analyzed the increasing wealth of genomic data to elucidate the SINE/LINE relationships in eukaryotic genomes, especially plants [34]. I proposed that the ancestral L1-clade LINE in the common ancestor of green plants may have used stringent RNA recognition to initiate reverse transcription. During the course of plant evolution, specific recognition of the RNA template may have been lost in a plant L1 lineage, as in mammals.

### 4.1. L1-Clade LINEs Are Predominant in the Genomes of Flowering Plants

**Figure 6:**
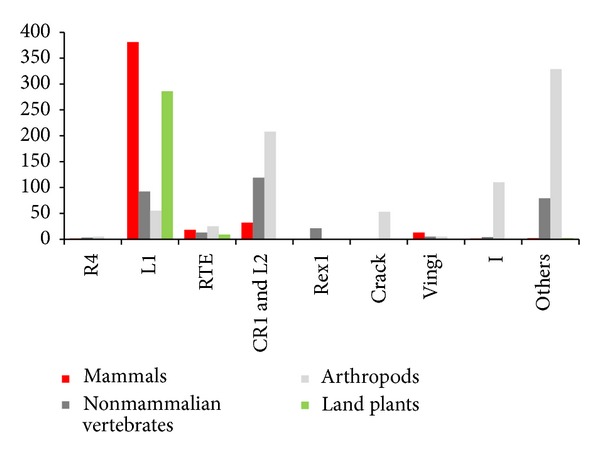
The number of LINE families belonging to each LINE clade according to biological taxa [34]. LINE clades in which the partner LINE of a SINE was identified are shown. The remaining clades are grouped as “Others” (Repbase 16.10); land plants: mostly flowering plants.

Figure 6 represents the number of LINEs belonging to each LINE clade according to biological taxa [34]. The L1 clade is the largest of all the clades, with L1-clade LINEs being predominant in mammals and land plants (mainly flowering plants). The genomes of flowering plants harbor almost exclusively L1-clade LINEs (RTE-clade LINEs are also found in several species). 

While a significant number of SINEs, more than half of which end in poly(A) repeats, have been identified in the genomes of flowering plants (Table 2) [34], only three SINE/LINE pairs have been discovered in their genomes, that is, maize ZmSINE2 and ZmSINE3 [66] and tobacco TS SINE [34]. Interestingly, many PPs have been reported in flowering plants [11, 70–73]. Since mammalian L1s are thought to recognize the 3′-poly(A) tail of RNA when forming PPs [43], it is possible that the plant LINE machinery is similar to that of mammalian L1s [68]; that is, plant L1-clade LINEs presumably recognize the 3′-poly(A) tail of RNA, thereby mobilizing SINEs with a poly(A) tail and mRNA. 

In accordance with this hypothesis, almost all L1-clade LINEs in flowering plants end in poly(A) repeats, while all RTE-clade LINEs end in (TTG)n or (TTGATG)n (Table 3) [34]. As for the exceptional cases of p-SINEs [74, 75] and Au-like SINEs [76–79], which end in poly(T) tracts (or a short stretch of T), it is possible that they are mobilized by unidentified partner LINEs that recognize a poly(U) repeat of RNA at the 3′-terminus.

### 4.2. Plant L1-Clade LINEs Consist of 3 Deeply Branching Lineages That Have Descended from the Common Ancestor of Monocots and Eudicots

Comprehensive phylogenetic analysis of L1-clade LINEs revealed three important points [34]. First, L1-clade LINEs from distinct taxa (i.e., land plants, green algae, and vertebrates) formed monophyletic groups. Statistical support for the monophyly of land plants and green algae was high, with bootstrap values of 100/82 and 97/83 (NJ/ML methods), respectively. The monophyly of vertebrate F and M lineages was not supported by the ML method. Second, the L1 lineages from these three taxa formed a monophyletic group (55/45; NJ/ML methods) among diverged LINE clades such as RTE and CR1. The Tx1 LINE, with a target-specific insertion, was also found in this clade, as observed in previous studies [26, 29, 30]. The Tx1 and vertebrate F lineage formed a monophyletic group with high confidence (94/85). Third, comparison with species phylogeny revealed that plant L1-clade LINEs consist of at least three deeply branching lineages that have descended from the common ancestor of monocots and eudicots (ME1–3). These 3 lineages must have arisen more than 130 mya, which is the approximate divergence of monocots and eudicots [80]. The history of plant L1 lineages is therefore reminiscent of that of vertebrate L1-clade LINEs, which are divided into several ancestral lineages (M and F/Tx1), one of which leads to mammalian L1s [28, 29].

### 4.3. A Conserved 3′-End Sequence with a Solid RNA Structure, as in Maize and Sorghum SINEs, Observed in One Plant L1 Lineage

**Figure 7:**
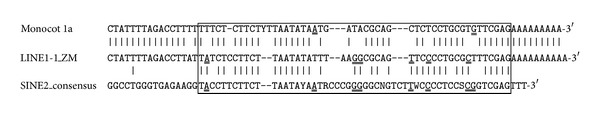
Sequence comparisons of the 3′-end sequences of L1-clade LINEs and monocot SINE families. The 3′-end sequences of the monocot 1a (consensus), LINE1-1_ZM, and SINE2 (consensus) were aligned [34]. Vertical lines and hyphens represent identical nucleotides and gaps, respectively. A conserved region between the LINEs and SINEs is boxed. R: A/G, Y: C/T, S: C/G, W: A/T, N: any nucleotide.

One monocot L1 lineage (monocot 1a in ME1) consisted of a large number of L1-clade LINEs that were identified mainly in the recently released maize and sorghum genomes. Moreover, one group of LINEs in this lineage retained a conserved 3′-end sequence [34]. The average pairwise divergence of this region (the last 45 nucleotides) among the LINEs was only 0.144 (standard error (SE), 0.043), whereas that for the entire sequence was 0.570 (SE, 0.012). Interestingly, maize SINEs (ZmSINE2 and ZmSINE3) with 3′-end sequences very similar to that of a LINE belonging to this group, LINE1-1_ZM, were reported recently [66]. I further revealed that several sorghum SINEs also possess similar 3′-end sequences [34]. Comparisons of the 3′-end sequences from these SINEs and LINEs revealed that part of the sequence (~50 nucleotides) is apparently related; presumably they were derived from a common ancestral L1 sequence (Figure 7) [34]. 

**Figure 8:**
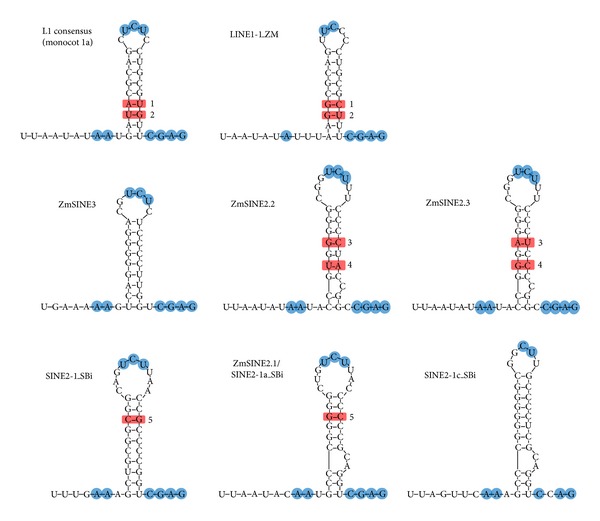
Secondary structure models for the 3′-end sequences of L1s and monocot SINEs. The putative transcripts form putative hairpin structures. Compensatory mutations (1–5) are shown by red rectangles. Conserved nucleotides are indicated by blue circles. The minimum free energy levels were −10.8 or −12.6 (kcal/mol) for L1s (monocot 1a and LINE1-1, resp.) and (−12.5)–(−13.7) for SINEs (ZmSINE2.3: −15.4 and SINE2-1c: −17.7). The structures were deduced using mfold [96].

Furthermore, the putative transcript from this region forms a putative hairpin structure (Figure 8) [34]. Compensatory mutations were observed in the stem-forming sequences, confirming a secondary structure. Several nucleotides were strongly conserved in the 3′-flanking region of the stem (5′-CGAG-3′) and in the loop (5′-UCU-3′), though the stem-forming nucleotides were variable. This stem-loop structure is commonly observed in the 3′-end sequences of LINEs and SINEs of the stringent type [38, 81, 82]. These results strongly suggest that, at least in this lineage, plant LINEs require a particular 3′-end sequence of the stringent type.

### 4.4. Origin of Stringent and Relaxed 3′-End Recognition of Plant L1-Clade LINEs

**Figure 9:**
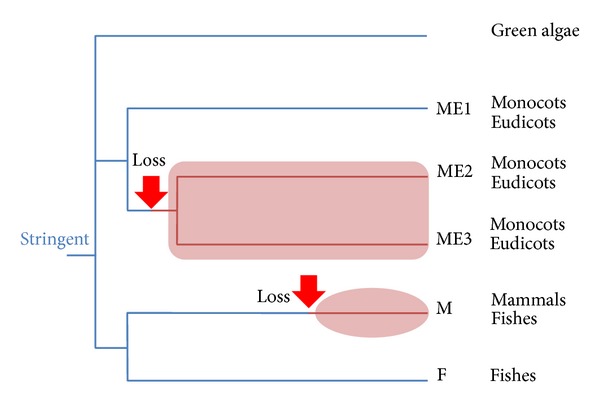
Proposed model for the 3′-end recognition of L1-clade LINEs. The ancestral L1-clade LINE in the ancestral green plant possessed a stringent, nonmammalian-type RNA recognition property. During the course of plant evolution, an L1 lineage lost the ability to recognize specifically the RNA template for reverse transcription, thereby introducing relaxed 3′-end recognition in land plants. ME1–3: plant L1 lineages; M, F: vertebrate L1 lineages.

The last example of a SINE/LINE pair in the L1-clade was found in a green alga. The 3′-end sequence (~80 nucleotides) of *Chlamydomonas* SINEX-3_CR [83] was very similar to that of L1-1_CR, both ending in poly(A) repeats [34]. Since land plants emerged from green algae [84], the following mechanism is proposed for the 3′-end recognition of plant L1-clade LINEs (Figure 9). 

It is possible that the ancestral L1-clade LINE in the genome of the common ancestor of green plants possessed stringent, nonmammalian-type RNA recognition properties. During the course of plant evolution, an L1 lineage then lost the ability to recognize specifically the RNA template for reverse transcription, thereby introducing relaxed 3′-end recognition in land (flowering) plants as well as in mammals. This model assumes that rigid sequence specificity was an ancestral state, although the timing of its loss might be subject to debate. Since horizontal transfer of LINEs between eukaryotes is rare [25, 85–87], the discontinuous distribution of L1-clade LINEs with low specificity (i.e., mammalian L1s and plant ME2/ME3) suggests a type of parallel evolution. 

The ancestral L1-clade LINE might have required the 3′-end sequence and the terminal poly(A) repeats. A few L1 lineages might then have lost their specific interaction with the 3′-UTR of the template RNA, retaining some role for the 3′-repeats. As shown in Table 3, most plant L1-clade LINEs, as well as mammalian L1s, have poly(A) repeats at their 3′-termini; however, 3′-poly(A) repeats are not necessarily a hallmark of relaxed 3′-end recognition. For example, although silkworm SART1, an R1-clade LINE, uses stringent-type recognition (its 3′-UTR is essential for retroposition) it ends in poly(A) repeats [37, 38], which are necessary for efficient and accurate retroposition [38]. Other LINEs end in repeating units other than poly(A); for example, the I element (I clade) ends in TAA repeats [88], while UnaL2 (L2) ends in TGTAA repeats, which are likely involved in template slippage during reverse transcription [36]. 

Alternatively, the ancestral L1-clade LINE may have possessed relaxed, mammalian-type RNA recognition properties. During the course of plant evolution, the L1 lineages of land plants (ME1) and green algae might then have gained specific stringent-type recognition of the RNA template. However, it is difficult to imagine that the molecular machinery for rigid sequence specificity, such as the particular conformation of the RNA-binding domain, has arisen independently under reduced constraints.

*In vivo* retroposition assays have been developed for several LINEs [36, 37, 39, 48]. Using such systems, it will be possible to verify these 2 models by evaluating the dispensability of the 3′-end sequence or poly(A) repeats in newly characterized L1 lineages such as plant ME1 and fish F. 

## 5. Concluding Remarks

L1 LINEs have contributed significantly to the architecture and evolution of mammalian genomes, whereas LTR retrotransposons are overwhelmingly found in certain flowering plants. Understanding the independent origins of flexible 3′-end recognition may help us to determine what distinguishes the fate of a retroposon in the eukaryotic genome and why it has succeeded so well in certain genomes [89–93].

## Figures and Tables

**Table 1:** Identification of SINE/LINE pairs [34].

SINE		Species	Promoter	LINE tail		Description of SINE/LINE pair
		**Mammals**				
MIR (CORE-SINEs: Ther-1, Mon-1)	[97–99]	All mammals	tRNA	L2	[15]	[100]
CORE-SINEs (MIR3/Ther-2)	[15, 99]	Mammals	tRNA	L3	[101]	[15, 34, 102]
CORE-SINEs (Mar-1/MAR1_MD)	[15, 99]	Marsupials	tRNA	RTE-3_MD	[15]	[15, 34, 102]
MAR4 (MAR4_MD, WALLSI3)	[15]	Opossum and wallaby, *Monodelphis domestica*, *Macropus eugenii *	(5′-end of RTE)	RTE-2 (MD, ME)	[15]	[15, 34]
RTESINE1	[15]	Opossum, *Monodelphis domestica *	(5′-end of RTE)	RTE-1_MD	[15]	[34]
Ped-1	[103]	Springhare, *Pedetes capensis* (*Rodentia*)	5S rRNA	BovB_Pca	[103]	[103]
Ped-2	[103]	Springhare, *Pedetes capensis* (*Rodentia*)	tRNA (ID SINE)	BovB_Pca	[103]	[103]
Bov-tA	[104, 105]	Ruminants	tRNA^Glu^	Bov-B	[105, 106]	[100, 104]
Bov-A2	[104, 105]	Ruminants	(5′-end of BovB)	Bov-B	[105, 106]	[104]
SINE2-1_EC	[15]	Horse, *Equus caballus *	tRNA	RTE-1_EC	[15]	[15, 34]
Afro SINEs (AFRO_LA, PSINE1)	[15, 107]	All Afrotherians	tRNA	RTE1 (LA, Pca)	[15]	[34, 103, 108]
RTE1-N1_LA	[15]	Elephant, *Loxodonta africana *	(5′-end of RTE)	RTE1_LA	[15]	[15]
SINE2-1_Pca	[15]	Hyrax, *Procavia capensis *	tRNA	RTE1_Pca	[15]	[15]

		**Birds and Reptiles**				
TguSINE1	[15]	Zebra finch, *Taeniopygia guttata *	tRNA^Ile^	CR1-X	[15]	[15]
Tortoise Pol III/SINE	[31, 109–111]	Tortoises and turtles, *Cryptodira *	tRNA^Lys^	PsCR1	[112]	[31, 113]
Sauria SINE	[114, 115]	Lizard, *Anolis carolinensis *	tRNA	Anolis Bov-B	[114]	[114]
Anolis SINE 2	[115]	Lizard, *Anolis carolinensis *	(Box A & B)	Anolis LINE 2	[115]	[115]
SINE2-1B_Acar/ SINE2-1_Acar	[15]	Lizard, *Anolis carolinensis *	tRNA	Vingi-2_Acar	[116]	[15, 34]

		**Amphibians**				
V-SINEs (SINE2-1_XT)	[15]	Frog, *Xenopus (Silurana) tropicalis *	tRNA	L2-4_XT (L2-3, L2-6, L2-2)	[15]	[15, 34]
CORE-SINEs (MIR_Xt)	[15]	Frog, *Xenopus (Silurana) tropicalis *	tRNA	L2-5_XT	[15]	[34]

		**Fish**				
Sma I	[117, 118]	Chum and pink salmon, *Oncorhynchus *	tRNA^Lys^	SalL2	[23]	[23, 31, 32]
Fok I	[118]	Charr, *Salvelinus *	tRNA^Lys^	SalL2	[23]	[23, 31, 32]
SlmI	[119]	All salmonids, *Salmonidae *	tRNA^Leu^	RSg-1	[120]	[119]
CORE-SINEs (Hpa I)	[102, 118]	All salmonids, *Salmonidae *	tRNA	RSg-1	[120]	[31]
CORE-SINEs (AFC, SINE2-1_AFC)	[102, 121, 122]	Cichlid fish, *Cichlidae *	tRNA	CiLINE2	[121]	[121]
CORE-SINEs (UnaSINE1, UnaSINE2)	[123]	Eel, *Anguilla japonica *	tRNA	UnaL2	[36, 123]	[36, 123]
HAmo SINE	[124]	Carp, *Cyprinidae *	tRNA	HAmoL2	[124]	[124]
DeuSINEs (AmnSINE1, SINE3)	[21, 125]	Mammals, chicken, zebrafish, catfish	5S rRNA	CR1-4_DR (CR1-7, CR1-9, CR1-13)	[15]	[21, 34, 125]
DeuSINEs (LmeSINE1, SacSINE1)	[125]	Coelacanth and dogfish shark, *Latimeria menadoensis*, *Squalus acanthias *	tRNA	CR1-4_DR-like	[15]	[125]
DeuSINEs (OS-SINE1)	[119, 125]	Salmon and trout, *Oncorhynchus*, *Salmo *	5S rRNA	RSg-1	[120]	[125]
V-SINEs (HE1)	[126, 127]	Sharks and rays, *M. manazo*, *S. torazame*, *H. japonicus*, *T. californica *	tRNA	HER1	[126]	[126]
V-SINEs (DANA)	[127–129]	Zebrafish, *Danio rerio *	tRNA	CR1-3DR/ZfL3	[15, 127]	[127]
V-SINEs (Lun1)	[127]	Lungfish, *Lepidosiren paradoxa *	tRNA	LfR1	[127]	[127]
SINEX-1_CM/SINE2-1_CM	[130]	Elephant shark, *Callorhinchus milii *	tRNA	CR1-2_CM	DQ524334	[34, 130]

		**Chordates**				
DeuSINEs (BflSINE1)	[125]	Amphioxus, *Branchiostoma floridae *	tRNA	Crack-16_BF	[15]	[34]

		**Deuterostomes**				
SURF1/SINE2-4c_SP	[15, 131]	Sea urchin, *Strongylocentrotus purpuratus *	tRNA	CR1-4_SP	[15]	[34]
DeuSINEs (SINE2-3_SP)	[15, 125]	Sea urchin, *Strongylocentrotus purpuratus *	tRNA	CR1Y_SP (CR1X_SP)	[15]	[15, 34]
SINE2-8_SP (SINE2-6, SINE2-4b)	[15]	Sea urchin, *Strongylocentrotus purpuratus *	tRNA	L2-1_SP/CR1-3_SP	[15]	[34]

		**Protostomes**				
Gecko	[132]	Mosquito, *Aedes aegypti *	tRNA	I-74_AAe (MosquI, I-58, I-59, I-62, I-64, I_Ele10, 14, 35, 37)	[15, 133]	[34, 132]

		**Eumetazoans**				
Nve-Nin-DC-SINE-1 (*1)	[134]	Sea anemone, *Nematostella vectensis *	tRNA	L2-22_NV	[15]	[134]
Nve-Nin-DC-SINE-2 (*1)	[134]	Sea anemone, *Nematostella vectensis *	tRNA	CR1-5_NV	[15]	[134]
Nve-Nin-DC-SINE-3 (*1)	[134]	Sea anemone, *Nematostella vectensis *	tRNA	CR1-15_NV	[15]	[134]
SINE2-1_NV	[15]	Sea anemone, *Nematostella vectensis *	tRNA	CR1-16_NV	[15]	[34]
SINE2-5_NV	[15]	Sea anemone, *Nematostella vectensis *	tRNA	Rex1-24_NV	[15]	[34]

		**Fungi**				
Mg-SINE	[135]	Rice blast fungus, *Magnaporthe grisea *	tRNA	MgL/MGR583	AF018033	[32, 34]
SINE2-1_BG	[15]	Powdery mildew fungus, *Blumeria graminis *	tRNA	Tad1-24_BG (HaTad1-3, 1-5)	[15]	[34]

		**Amoebozoa**				
EdSINE1 (SINE-lile)	[136]	Amoeba, *Entamoeba dispar *	Unknown	R4-1_ED	[15]	[34]
R4-N1_ED (SINE-lile)	[15]	Amoeba, *Entamoeba dispar *	Unknown	R4-1_ED	[15]	[34]
EhLSINE1/ehapt2 (SINE-lile)	[137, 138]	Amoeba, *Entamoeba histolytica *	Unknown	EhLINE1/EhRLE1	[30, 137]	[137]
EhLSINE2 (SINE-like)	[137]	Amoeba, *Entamoeba histolytica *	Unknown	EhLINE2/EhRLE3	[30, 137]	[137]

		**Land plants**				
TS	[35]	Tobacco, *Nicotiana tabacum *	tRNA	RTE-1_Stu	[15]	[34]
ZmSINE2/SINE2_SBi	[66]	Maize, *Zea mays*; Sorghum, *Sorghum bicolor *	tRNA	LINE1-1_ZM	[15]	[34, 66]
ZmSINE3	[66]	Maize, *Zea mays *	tRNA	LINE1-1_ZM	[15]	[66]

		**Green algae**				
SINEX-1_CR	[15, 83]	*Chlamydomonas reinhardtii *	Unknown	RandI-2/ DualenCr3	[15, 139]	[34, 83]
SINEX-2_CR	[15, 83]	*Chlamydomonas reinhardtii *	Unknown	RandI-2 (RandI-3)	[15, 139]	[83]
SINEX-3_CR	[15, 83]	*Chlamydomonas reinhardtii *	tRNA	L1-1_CR	[15]	[15, 83]
SINEX-4_CR	[15, 83]	*Chlamydomonas reinhardtii *	Unknown	RandI-2 (RandI-3)	[15, 139]	[34, 83]
SINEX-5_CR/SINEX-6_CR	[83]	*Chlamydomonas reinhardtii *	tRNA	RandI-5	[15]	[83]

(*1) Subfamilies.

**Table 2:** 3′-Repeats of plant SINE families [34].

SINE	Species	3′-Repeat	LINE tail	Reference for SINEs
	**Green algae**			
SINEX-1_CR	*Chlamydomonas reinhardtii *	(ATT)*n*	RandI-2/DualenCr3	[83]
SINEX-2_CR	*Chlamydomonas reinhardtii *	(CTTT)*n*	RandI-2 (RandI-3)	[83]
SINEX-3_CR	*Chlamydomonas reinhardtii *	(A)*n*	L1-1_CR	[83]
SINEX-4_CR	*Chlamydomonas reinhardtii *	(ATT)*n*	RandI-2 (RandI-3)	[83]
SINEX-5_CR/SINEX-6_CR	*Chlamydomonas reinhardtii *	(ATT)*n*	RandI-5	[83]

	**Seed plants**			
Au	Angiosperms and a gymnosperm	(T)2–5	Nd	[76–79]
ZmSINE1 (Au-like)	*Zea mays *	(T)*n*	Nd	[66]
SINE2-1_ZM (Au-like)	*Zea mays *	(T)3	Nd	[15]
SINE-5_Mad (Au-like)	*Malus* x *domestica *	(T)3	Nd	[15]

	**Monocots**			
p-SINE1	*Oryza sativa *	(T)*n*	Nd	[74]
p-SINE2	*Oryza sativa *	(T)*n*	Nd	[75]
p-SINE3	*Oryza sativa *	(T)*n*	Nd	[75]
ZmSINE2.1*/SINE2-1a_SBi	*Zea mays*, *Sorghum bicolor *	(T)*n*	LINE1-1_ZM	[15, 66]
ZmSINE2.2*	*Zea mays *	(T)*n*	LINE1-1_ZM	[66]
ZmSINE2.3*	*Zea mays *	(T)*n*	LINE1-1_ZM	[66]
SINE2-1_SBi (ZmSINE2-like)	*Sorghum bicolor *	(T)*n*	LINE1-1_ZM	[15]
SINE2-1c_SBi (ZmSINE2-like)	*Sorghum bicolor *	(T)*n*	LINE1-1_ZM	[15]
ZmSINE3	*Zea mays *	(A)*n*	LINE1-1_ZM	[66]
OsSN1/F524	*Oryza sativa *	(A)*n*	Nd	[140]
OsSN2/SINE2-12_SBi	*Oryza sativa*, *Sorghum bicolor *	(A)*n*	Nd	[15, 140]
OsSN3	*Oryza sativa *	(A)*n*	Nd	[140]
SINE9_OS/SINE2-11_SBi (OsSN-like)	*Oryza sativa*, *Sorghum bicolor *	(A)*n*	Nd	[15]

	**Eudicots**			
TS	*Nicotiana tabacum *	(TTG)*n*	RTE-1_STu	[35]
SB1-15 (S1/AtSN/RAthE/BoS)	*Arabidopsis thaliana*,* Brassicaceae* (*Cruciferae*)	(A)*n*	Nd	[68, 69, 141–144]
LJ_SINE-1	*Lotus japonicus *	(A)*n*	Nd	[145]
LJ_SINE-2	*Lotus japonicus *	(A)*n*	Nd	[145]
LJ_SINE-3	*Lotus japonicus *	(A)*n*	Nd	[145]
MT_SINE-1	*Medicago truncatula *	(A)*n*	Nd	[145]
MT_SINE-2	*Medicago truncatula *	(A)*n*	Nd	[145]
MT_SINE-3	*Medicago truncatula *	(A)*n*	Nd	[145]
SINE-1_Mad	*Malus* x *domestica *	(A)*n*	Nd	[15]
SINE-2_Mad	*Malus* x *domestica *	(A)*n*	Nd	[15]
SINE-4_Mad	*Malus* x *domestica *	(A)*n*	Nd	[15]
SINE2-1_PTr	*Populus trichocarpa *	(A)*n*	Nd	[15]
SINE2-2_PTr	*Populus trichocarpa *	(A)*n*	Nd	[15]

*subfamilies. Nd: no data.

**Table 3:** 3′-Repeats of plant LINE families [34].

Species	LINE clade	Families	3′-Repeat		
			(A)*n*	Other repeats	None
Flowering plants	L1	233	224	0	9
	RTE	7	0	7^∗b^	0

	L1	15	2^∗a^	8^∗c^	5
Green algae	RandI	8	0	8^∗d^	0
	RTEX	6	0	6^∗e^	0

^∗a^L1-1_CR (*Chlamydomonas*), Zepp (*Chlorella*).

^∗b^(TTG)*n*, (TTGATG)*n*.

^∗c^(CATA)*n*, (CA)*n*, (CAA)*n*, (TAA)*n*.

^∗d^(ATT)*n*, (CTATTT)*n*.

^∗e^(CA)*n*, (CAA)*n*, (CCAT)*n*, (ACAATG)*n*, (CTTGTAA)*n*.

## References

[1] Ohno S (1970). *Evolution by Gene Duplication*.

[2] Long M, Betrán E, Thornton K, Wang W (2003). The origin of new genes: glimpses from the young and old. *Nature Reviews Genetics*.

[3] Babushok DV, Ostertag EM, Kazazian HH (2007). Current topics in genome evolution: molecular mechanisms of new gene formation. *Cellular and Molecular Life Sciences*.

[4] Charon C, Bruggeman Q, Thareau V, Henry Y (2012). Gene duplication within the green lineage: the case of *TEL* genes. *Journal of Experimental Botany*.

[5] Vanin EF (1985). Processed pseudogenes: characteristics and evolution. *Annual Review of Genetics*.

[6] Weiner AM, Deininger PL, Efstratiadis A (1986). Nonviral retroposons: genes, pseudogenes, and transposable elements generated by the reverse flow of genetic information. *Annual Review of Biochemistry*.

[7] Yu Z, Morais D, Ivanga M, Harrison PM (2007). Analysis of the role of retrotransposition in gene evolution in vertebrates. *BMC Bioinformatics*.

[8] Liu Y-J, Zheng D, Balasubramanian S (2009). Comprehensive analysis of the pseudogenes of glycolytic enzymes in vertebrates: the anomalously high number of GAPDH pseudogenes highlights a recent burst of retrotrans-positional activity. *BMC Genomics*.

[9] Brosius J (1999). RNAs from all categories generate retrosequences that may be exapted as novel genes or regulatory elements. *Gene*.

[10] Kaessmann H, Vinckenbosch N, Long M (2009). RNA-based gene duplication: mechanistic and evolutionary insights. *Nature Reviews Genetics*.

[11] Sakai H, Mizuno H, Kawahara Y (2011). Retrogenes in rice (*Oryza sativa* L. ssp. *japonica*) exhibit correlated expression with their source genes. *Genome Biology and Evolution*.

[12] Ciomborowska J, Rosikiewicz W, Szklarczyk D, Makałowski W, Makałowska I (2013). “Orphan” retrogenes in the human genome. *Molecular Biology and Evolution*.

[13] Kazazian HH (2004). Mobile elements: drivers of genome evolution. *Science*.

[14] Brosius J (1991). Retroposons—seeds of evolution. *Science*.

[15] Jurka J, Kapitonov VV, Pavlicek A, Klonowski P, Kohany O, Walichiewicz J (2005). Repbase Update, a database of eukaryotic repetitive elements. *Cytogenetic and Genome Research*.

[16] Luan DD, Korman MH, Jakubczak JL, Eickbush TH (1993). Reverse transcription of R2Bm RNA is primed by a nick at the chromosomal target site: a mechanism for non-LTR retrotransposition. *Cell*.

[17] Luan DD, Eickbush TH (1995). RNA template requirements for target DNA-primed reverse transcription by the R2 retrotransposable element. *Molecular and Cellular Biology*.

[18] Goodier JL, Kazazian HH (2008). Retrotransposons revisited: the restraint and rehabilitation of parasites. *Cell*.

[19] Cost GJ, Feng Q, Jacquier A, Boeke JD (2002). Human L1 element target-primed reverse transcription *in vitro*. *The EMBO Journal*.

[20] Okada N (1991). SINEs: short interspersed repeated elements of the eukaryotic genome. *Trends in Ecology and Evolution*.

[21] Kapitonov VV, Jurka J (2003). A novel class of SINE elements derived from 5S rRNA. *Molecular Biology and Evolution*.

[22] Vassetzky NS, Kramerov DA (2013). SINEBase: a database and tool for SINE analysis. *Nucleic Acids Research*.

[23] Ohshima K, Okada N (2005). SINEs and LINEs: symbionts of eukaryotic genomes with a common tail. *Cytogenetic and Genome Research*.

[24] Schmitz J, Zemann A, Churakov G (2008). Retroposed SNOfall—a mammalian-wide comparison of platypus snoRNAs. *Genome Research*.

[25] Malik HS, Burke WD, Eickbush TH (1999). The age and evolution of non-LTR retrotransposable elements. *Molecular Biology and Evolution*.

[26] Kapitonov VV, Tempel S, Jurka J (2009). Simple and fast classification of non-LTR retrotransposons based on phylogeny of their RT domain protein sequences. *Gene*.

[27] Furano AV, Duvernell DD, Boissinot S (2004). L1 (LINE-1) retrotransposon diversity differs dramatically between mammals and fish. *Trends in Genetics*.

[28] Kordiš D, Lovšin N, Gubenšek F (2006). Phylogenomic analysis of the L1 retrotransposons in Deuterostomia. *Systematic Biology*.

[29] Ichiyanagi K, Nishihara H, Duvernell DD, Okada N (2007). Acquisition of endonuclease specificity during evolution of L1 retrotransposon. *Molecular Biology and Evolution*.

[30] Kojima KK, Fujiwara H (2004). Cross-genome screening of novel sequence-specific Non-LTR retrotransposons: various multicopy RNA genes and microsatellites are selected as targets. *Molecular Biology and Evolution*.

[31] Ohshima K, Hamada M, Terai Y, Okada N (1996). The 3′ ends of tRNA-derived short interspersed repetitive elements are derived from the 3′ ends of long interspersed repetitive elements. *Molecular and Cellular Biology*.

[32] Okada N, Hamada M, Ogiwara I, Ohshima K (1997). SINEs and LINEs share common 3′ sequences: a review. *Gene*.

[33] Weiner AM (2002). SINEs and LINEs: the art of biting the hand that feeds you. *Current Opinion in Cell Biology*.

[34] Ohshima K (2012). Parallel relaxation of stringent RNA recognition in plant and mammalian L1 retrotransposons. *Molecular Biology and Evolution*.

[35] Yoshioka Y, Matsumoto S, Kojima S, Ohshima K, Okada N, Machida Y (1993). Molecular characterization of a short interspersed repetitive element from tobacco that exhibits sequence homology to specific tRNAs. *Proceedings of the National Academy of Sciences of the United States of America*.

[36] Kajikawa M, Okada N (2002). LINEs mobilize SINEs in the eel through a shared 3′ sequence. *Cell*.

[37] Takahashi H, Fujiwara H (2002). Transplantation of target site specificity by swapping the endonuclease domains of two LINEs. *The EMBO Journal*.

[38] Osanai M, Takahashi H, Kojima KK, Hamada M, Fujiwara H (2004). Essential motifs in the 3′ untranslated region required for retrotransposition and the precise start of reverse transcription in non-long-terminal-repeat retrotransposon SART1. *Molecular and Cellular Biology*.

[39] Anzai T, Osanai M, Hamada M, Fujiwara H (2005). Functional roles of 3′-terminal structures of template RNA during *in vivo* retrotransposition of non-LTR retrotransposon, R1Bm. *Nucleic Acids Research*.

[40] Feng Q, Moran JV, Kazazian HH, Boeke JD (1996). Human L1 retrotransposon encodes a conserved endonuclease required for retrotransposition. *Cell*.

[41] Jurka J (1997). Sequence patterns indicate an enzymatic involvement in integration of mammalian retroposons. *Proceedings of the National Academy of Sciences of the United States of America*.

[42] Pavlíček A, Pačes J, Elleder D, Hejnar J (2002). Processed pseudogenes of human endogenous retroviruses generated by LINEs: their integration, stability, and distribution. *Genome Research*.

[43] Esnault C, Maestre J, Heidmann T (2000). Human LINE retrotransposons generate processed pseudogenes. *Nature Genetics*.

[44] Wei W, Gilbert N, Ooi SL (2001). Human L1 retrotransposition: *cis* preference versus *trans* complementation. *Molecular and Cellular Biology*.

[45] Roy-Engel AM, Salem A-H, Oyeniran OO (2002). Active Alu element “A-tails”: size does matter. *Genome Research*.

[46] Dewannieux M, Esnault C, Heidmann T (2003). LINE-mediated retrotransposition of marked Alu sequences. *Nature Genetics*.

[47] Kroutter EN, Belancio VP, Wagstaff BJ, Roy-Engel AM (2009). The RNA polymerase dictates ORF1 requirement and timing of LINE and SINE retrotransposition. *PLoS Genetics*.

[48] Moran JV, Holmes SE, Naas TP, DeBerardinis RJ, Boeke JD, Kazazian HH (1996). High frequency retrotransposition in cultured mammalian cells. *Cell*.

[49] Moran JV, DeBerardinis RJ, Kazazian HH (1999). Exon shuffling by L1 retrotransposition. *Science*.

[50] Ohshima K, Hattori M, Yada T, Gojobori T, Sakaki Y, Okada N (2003). Whole-genome screening indicates a possible burst of formation of processed pseudogenes and Alu repeats by particular L1 subfamilies in ancestral primates. *Genome Biology*.

[51] Boeke JD (1997). LINEs and *Alus*—the polyA connection. *Nature genetics*.

[52] Schmitz J, Churakov G, Zischler H, Brosius J (2004). A novel class of mammalian-specific tailless retropseudogenes. *Genome Research*.

[53] Zhang Z, Harrison PM, Liu Y, Gerstein M (2003). Millions of years of evolution preserved: a comprehensive catalog of the processed pseudogenes in the human genome. *Genome Research*.

[54] Marques AC, Dupanloup I, Vinckenbosch N, Reymond A, Kaessmann H (2005). Emergence of young human genes after a
burst of retroposition in primates. *PLoS Biology*.

[55] Sakai H, Koyanagi KO, Imanishi T, Itoh T, Gojobori T (2007). Frequent emergence and functional resurrection of processed pseudogenes in the human and mouse genomes. *Gene*.

[56] Wagstaff BJ, Kroutter EN, Derbes RS, Belancio VP, Roy-Engel AM (2013). Molecular reconstruction of extinct LINE-1 elements and their interaction with nonautonomous elements. *Molecular Biology and Evolution*.

[57] Burki F, Kaessmann H (2004). Birth and adaptive evolution of a hominoid gene that supports high neurotransmitter flux. *Nature Genetics*.

[58] Rosso L, Marques AC, Weier M (2008). Birth and rapid subcellular adaptation of a hominoid-specific CDC14 protein. *PLoS Biology*.

[59] Babushok DV, Ohshima K, Ostertag EM (2007). A novel testis ubiquitin-binding protein gene arose by exon shuffling in hominoids. *Genome Research*.

[60] Zhang Y, Lu S, Zhao S, Zheng X, Long M, Wei L (2009). Positive selection for themale functionality of a co-retroposed gene in the hominoids. *BMC Evolutionary Biology*.

[61] Ohshima K, Igarashi K (2010). Inference for the initial stage of domain shuffling: tracing the evolutionary fate of the *PIPSL* retrogene in hominoids. *Molecular Biology and Evolution*.

[62] Harrison PM, Zheng D, Zhang Z, Carriero N, Gerstein M (2005). Transcribed processed pseudogenes in the human genome: an intermediate form of expressed retrosequence lacking protein-coding ability. *Nucleic Acids Research*.

[63] Vinckenbosch N, Dupanloup I, Kaessmann H (2006). Evolutionary fate of retroposed gene copies in the human genome. *Proceedings of the National Academy of Sciences of the United States of America*.

[64] Baertsch R, Diekhans M, Kent WJ, Haussler D, Brosius J (2008). Retrocopy contributions to the evolution of the human genome. *BMC Genomics*.

[65] Kojima KK, Okada N (2009). mRNA retrotransposition coupled with 5′ inversion as a possible source of new genes. *Molecular Biology and Evolution*.

[66] Baucom RS, Estill JC, Chaparro C (2009). Exceptional diversity, non-random distribution, and rapid evolution of retroelements in the B73 maize genome. *PLoS Genetics*.

[67] Noma K, Ohtsubo H, Ohtsubo E (2000). *ATLN* elements, LINEs from *Arabidopsis thaliana*: identification and characterization. *DNA Research*.

[68] Lenoir A, Lavie L, Prieto J-L (2001). The evolutionary origin and genomic organization of SINEs in *Arabidopsis thaliana*. *Molecular Biology and Evolution*.

[69] Myouga F, Tsuchimoto S, Noma K, Ohtsubo H, Ohtsubo E (2001). Identification and structural analysis of SINE elements in the *Arabidopsis thaliana* genome. *Genes and Genetic Systems*.

[70] Faris J, Sirikhachornkit A, Haselkorn R, Gill B, Gornicki P (2001). Chromosome mapping and phylogenetic analysis of the cytosolic acetyl-CoA carboxylase loci in wheat. *Molecular Biology and Evolution*.

[71] Zhang Y, Wu Y, Liu Y, Han B (2005). Computational identification of 69 retroposons in Arabidopsis. *Plant Physiology*.

[72] Benovoy D, Drouin G (2006). Processed pseudogenes, processed genes, and spontaneous mutations in the *Arabidopsis *genome. *Journal of Molecular Evolution*.

[73] Nurhayati N, Gondé D, Ober D (2009). Evolution of pyrrolizidine alkaloids in *Phalaenopsis* orchids and other monocotyledons: identification of deoxyhypusine synthase, homospermidine synthase and related pseudogenes. *Phytochemistry*.

[74] Mochizuki K, Umeda M, Ohtsubo H, Ohtsubo E (1992). Characterization of a plant SINE, p-SINE*1*, in rice genomes. *Japanese Journal of Genetics*.

[75] Xu J-H, Osawa I, Tsuchimoto S, Ohtsubo E, Ohtsubo H (2005). Two new SINE elements, *p-SINE2* and *p-SINE3*, from rice. *Genes and Genetic Systems*.

[76] Yasui Y, Nasuda S, Matsuoka Y, Kawahara T (2001). The Au family, a novel short interspersed element (SINE) from *Aegilops umbellulata*. *Theoretical and Applied Genetics*.

[77] Fawcett JA, Kawahara T, Watanabe H, Yasui Y (2006). A SINE family widely distributed in the plant kingdom and its evolutionary history. *Plant Molecular Biology*.

[78] Yagi E, Akita T, Kawahara T (2011). A novel Au SINE sequence found in a gymnosperm. *Genes and Genetic Systems*.

[79] Shu Y, Li Y, Bai X (2011). Identification and characterization of a new member of the SINE Au retroposon family (GmAu1) in the soybean, *Glycine max* (L.) Merr., genome and its potential application. *Plant Cell Reports*.

[80] Moore MJ, Bell CD, Soltis PS, Soltis DE (2007). Using plastid genome-scale data to resolve enigmatic relationships among basal angiosperms. *Proceedings of the National Academy of Sciences of the United States of America*.

[81] Mathews DH, Banerjee AR, Luan DD, Eickbush TH, Turner DH (1997). Secondary structure model of the RNA recognized by the reverse transcriptase from the R2 retrotransposable element. *RNA*.

[82] Nomura Y, Kajikawa M, Baba S (2006). Solution structure and functional importance of a conserved RNA hairpin of eel LINE UnaL2. *Nucleic Acids Research*.

[83] Cognat V, Deragon J-M, Vinogradova E, Salinas T, Remacle C, Maréchal-Drouard L (2008). On the evolution and expression of *Chlamydomonas reinhardtii* nucleus-encoded transfer RNA genes. *Genetics*.

[84] Karol KG, McCourt RM, Cimino MT, Delwiche CF (2001). The closest living relatives of land plants. *Science*.

[85] Kidwell MG, Lisch D (1997). Transposable elements as sources of variation in animals and plants. *Proceedings of the National Academy of Sciences of the United States of America*.

[86] Kordiš D, Gubenšek F (1998). Unusual horizontal transfer of a long interspersed nuclear element between distant vertebrate classes. *Proceedings of the National Academy of Sciences of the United States of America*.

[87] Walsh AM, Kortschak RD, Gardner MG, Bertozzi T, Adelson DL (2013). Widespread horizontal transfer of retrotransposons. *Proceedings of the National Academy of Sciences of the United States of America*.

[88] Chambeyron S, Bucheton A, Busseau I (2002). Tandem UAA repeats at the 3′-end of the transcript are essential for the precise initiation of reverse transcription of the I factor in *Drosophila melanogaster*. *Journal of Biological Chemistry*.

[89] Komatsu M, Shimamoto K, Kyozuka J (2003). Two-step regulation and continuous retrotransposition of the rice LINE-type retrotransposon *Karma*. *Plant Cell*.

[90] Zhang X, Wessler SR (2004). Genome-wide comparative analysis of the transposable elements in the related species *Arabidopsis thaliana* and *Brassica oleracea*. *Proceedings of the National Academy of Sciences of the United States of America*.

[91] Yamashita H, Tahara M (2006). A LINE-type retrotransposon active in meristem stem cells causes heritable transpositions in the sweet potato genome. *Plant Molecular Biology*.

[92] Heitkam T, Schmidt T (2009). BNR—a LINE family from *Beta vulgaris*—contains a RRM domain in open reading frame 1 and defines a L1 sub-clade present in diverse plant genomes. *Plant Journal*.

[93] Hollister JD, Smith LM, Guo Y-L, Ott F, Weigel D, Gaut BS (2011). Transposable elements and small RNAs contribute to gene expression divergence between *Arabidopsis thaliana* and *Arabidopsis lyrata*. *Proceedings of the National Academy of Sciences of the United States of America*.

[94] Khazina E, Truffault V, Büttner R, Schmidt S, Coles M, Weichenrieder O (2011). Trimeric structure and flexibility of the L1ORF1 protein in human L1 retrotransposition. *Nature Structural and Molecular Biology*.

[95] Smerdon SJ, Jäger J, Wang J (1994). Structure of the binding site for nonnucleoside inhibitors of the reverse transcriptase of human immunodeficiency virus type 1. *Proceedings of the National Academy of Sciences of the United States of America*.

[96] Zuker M (2003). Mfold web server for nucleic acid folding and hybridization prediction. *Nucleic Acids Research*.

[97] Smit AFA, Riggs AD (1995). MIRs are classic, tRNA-derived SINEs that amplified before the mammalian radiation. *Nucleic Acids Research*.

[98] Jurka J, Zietkiewicz E, Labuda D (1995). Ubiquitous mammalian-wide interspersed repeats (MIRs) are molecular fossils from the mesozoic era. *Nucleic Acids Research*.

[99] Gilbert N, Labuda D (2000). Evolutionary inventions and continuity of CORE-SINEs in mammals. *Journal of Molecular Biology*.

[100] Smit AFA (1996). The origin of interspersed repeats in the human genome. *Current Opinion in Genetics and Development*.

[101] Kapitonov VV, Jurka J (2003). The esterase and PHD domains in CR1-like non-LTR retrotransposons. *Molecular Biology and Evolution*.

[102] Gilbert N, Labuda D (1999). CORE-SINEs: eukaryotic short interspersed retroposing elements with common sequence motifs. *Proceedings of the National Academy of Sciences of the United States of America*.

[103] Gogolevsky KP, Vassetzky NS, Kramerov DA (2008). Bov-B-mobilized SINEs in vertebrate genomes. *Gene*.

[104] Okada N, Hamada M (1997). The 3′ ends of tRNA-derived SINEs originated from the 3′ ends of LINEs: a new example from the bovine genome. *Journal of Molecular Evolution*.

[105] Lenstra JA, van Boxtel JA, Zwaagstra KA, Schwerin M (1993). Short interspersed nuclear element (SINE) sequences of the Bovidae. *Animal Genetics*.

[106] Szemraj J, Płucienniczak G, Jaworski J, Płucienniczak A (1995). Bovine *Alu*-like sequences mediate transposition of a new site-specific retroelement. *Gene*.

[107] Nikaido M, Nishihara H, Hukumoto Y, Okada N (2003). Ancient SINEs from African endemic mammals. *Molecular Biology and Evolution*.

[108] Gilbert C, Pace JK, Waters PD (2008). Target site analysis of RTE1_LA and its AfroSINE partner in the elephant genome. *Gene*.

[109] Endoh H, Okada N (1986). Total DNA transcription *in vitro*: a procedure to detect highly repetitive and transcribable sequences with tRNA-like structures. *Proceedings of the National Academy of Sciences of the United States of America*.

[110] Endoh H, Nagahashi S, Okada N (1990). A highly repetitive and transcribable sequence in the tortoise genome is probably a retroposon. *European Journal of Biochemistry*.

[111] Sasaki T, Takahashi K, Nikaido M, Miura S, Yasukawa Y, Okada N (2004). First application of the SINE (Short Interspersed Repetitive Element) method to infer phylogenetic relationships in reptiles: an example from the turtle superfamily testudinoidea. *Molecular Biology and Evolution*.

[112] Kajikawa M, Ohshima K, Okada N (1997). Determination of the entire sequence of turtle CR1: the first open reading frame of the turtle CR1 element encodes a protein with a novel zinc finger motif. *Molecular Biology and Evolution*.

[113] Vandergon TL, Reitman M (1994). Evolution of chicken repeat 1 (CR1) elements: evidence for ancient subfamilies and multiple progenitors. *Molecular Biology and Evolution*.

[114] Piskurek O, Austin CC, Okada N (2006). Sauria SINEs: novel short interspersed retroposable elements that are widespread in reptile genomes. *Journal of Molecular Evolution*.

[115] Piskurek O, Nishihara H, Okada N (2009). The evolution of two partner LINE/SINE families and a full-length chromodomain-containing *Ty3/Gypsy* LTR element in the first reptilian genome of *Anolis carolinensis*. *Gene*.

[116] Kojima KK, Kapitonov VV, Jurka J (2011). Recent expansion of a new *Ingi*-related clade of *Vingi* non-LTR retrotransposons in hedgehogs. *Molecular Biology and Evolution*.

[117] Matsumoto K-I, Murakami K, Okada N (1986). Gene for lysine tRNA_1_ may be a progenitor of the highly repetitive and transcribable sequences present in the salmon genome. *Proceedings of the National Academy of Sciences of the United States of America*.

[118] Kido Y, Aono M, Yamaki T (1991). Shaping and reshaping of salmonid genomes by amplification of tRNA-derived retroposons during evolution. *Proceedings of the National Academy of Sciences of the United States of America*.

[119] Matveev V, Nishihara H, Okada N (2007). Novel SINE families from salmons validate *Parahucho* (Salmonidae) as a distinct genus and give evidence that SINEs can incorporate LINE-related 3′-tails of other SINEs. *Molecular Biology and Evolution*.

[120] Winkfein RJ, Moir RD, Krawetz SA, Blanco J, States JC, Dixon GH (1988). A new family of repetitive, retroposon-like sequences in the genome of the rainbow trout. *European Journal of Biochemistry*.

[121] Terai Y, Takahashi K, Okada N (1998). SINE cousins: the 3′-end tails of the two oldest and distantly related families of SINEs are descended from the 3′ ends of LINEs with the same genealogical origin. *Molecular Biology and Evolution*.

[122] Takahashi K, Terai Y, Nishida M, Okada N (1998). A novel family of short interspersed repetitive elements (SINEs) from cichlids: the patterns of insertion of SINEs at orthologous loci support the proposed monophyly of four major groups of cichlid fishes in Lake Tanganyika. *Molecular Biology and Evolution*.

[123] Kajikawa M, Ichiyanagi K, Tanaka N, Okada N (2005). Isolation and characterization of active LINE and SINEs from the eel. *Molecular Biology and Evolution*.

[124] Tong C, Guo B, He S (2009). Bead-probe complex capture a couple of SINE and LINE family from genomes of two closely related species of East Asian cyprinid directly using magnetic separation. *BMC Genomics*.

[125] Nishihara H, Smit AFA, Okada N (2006). Functional noncoding sequences derived from SINEs in the mammalian genome. *Genome Research*.

[126] Ogiwara I, Miya M, Ohshima K, Okada N (1999). Retropositional parasitism of SINEs on LINEs: identification of SINEs and LINEs in elasmobranchs. *Molecular Biology and Evolution*.

[127] Ogiwara I, Miya M, Ohshima K, Okada N (2002). V-SINEs: a new superfamily of vertebrate SINEs that are widespread in vertebrate genomes and retain a strongly conserved segment within each repetitive unit. *Genome Research*.

[128] Izsvák Z, Ivics Z, Garcia-Estefania D, Fahrenkrug SC, Hackett PB (1996). DANA elements: a family of composite, tRNA-derived short interspersed DNA elements associated with mutational activities in zebrafish (*Danio rerio*). *Proceedings of the National Academy of Sciences of the United States of America*.

[129] Shimoda N, Chevrette M, Ekker M, Kikuchi Y, Hotta Y, Okamoto H (1996). *Mermaid*, a family of short interspersed repetitive elements, is useful for zebrafish genome mapping. *Biochemical and Biophysical Research Communications*.

[130] Venkatesh B, Kirkness EF, Loh Y-H (2007). Survey sequencing and comparative analysis of the elephant shark (*Callorhinchus milii*) genome. *PLoS Biology*.

[131] Nisson PE, Hickey RJ, Boshar MF, Crain WR (1988). Identification of a repeated sequence in the genome of the sea urchin which is traescribed by RNA polymerase III and contains the features of a retroposon. *Nucleic Acids Research*.

[132] Tu Z, Li S, Mao C (2004). The changing tails of a novel short interspersed element in *Aedes aegypti*: genomic evidence for slippage retrotransposition and the relationship between 3′ tandem repeats and the poly(dA) tail. *Genetics*.

[133] Tu Z, Hill JJ (1999). *MosquI*, a novel family of mosquito retrotransposons distantly related to the *Drosophila* I factors, may consist of elements of more than one origin. *Molecular Biology and Evolution*.

[134] Piskurek O, Jackson DJ (2011). Tracking the ancestry of a deeply conserved eumetazoan SINE domain. *Molecular Biology and Evolution*.

[135] Kachroo P, Leong SA, Chattoo BB (1995). Mg-SINE: a short interspersed nuclear element from the rice blast fungus, *Magnaporthe grisea*. *Proceedings of the National Academy of Sciences of the United States of America*.

[136] Shire AM, Ackers JP (2007). SINE elements of *Entamoeba dispar*. *Molecular and Biochemical Parasitology*.

[137] Van Dellen K, Field J, Wang Z, Loftus B, Samuelson J (2002). LINEs and SINE-like elements of the protist *Entamoeba histolytica*. *Gene*.

[138] Willhoeft U, Buß H, Tannich E (2002). The abundant polyadenylated transcript 2 DNA sequence of the pathogenic protozoan parasite *Entamoeba histolytica* represents a nonautonomous non-long-terminal-repeat retrotransposon-like element which is absent in the closely related nonpathogenic species *Entamoeba dispar*. *Infection and Immunity*.

[139] Kojima KK, Fujiwara H (2005). An extraordinary retrotransposon family encoding dual endonucleases. *Genome Research*.

[140] Tsuchimoto S, Hirao Y, Ohtsubo E, Ohtsubo H (2008). New SINE families from rice, *OsSN*, with poly(A) at the 3′ ends. *Genes and Genetic Systems*.

[141] Deragon J-M, Landry BS, Pélissier T, Tutois S, Tourmente S, Picard G (1994). An analysis of retroposition in plants based on a family of SINEs from *Brassica napus*. *Journal of Molecular Evolution*.

[142] Lenoir A, Pélissier T, Bousquet-Antonelli C, Deragon JM (2005). Comparative evolution history of SINEs in *Arabidopsis thaliana* and *Brassica oleracea*: evidence for a high rate of SINE loss. *Cytogenetic and Genome Research*.

[143] Zhang X, Wessler SR (2005). *BoS*: a large and diverse family of short interspersed elements (SINEs) in *Brassica oleracea*. *Journal of Molecular Evolution*.

[144] Deragon J-M, Zhang X (2006). Short interspersed elements (SINEs) in plants: origin, classification, and use as phylogenetic markers. *Systematic Biology*.

[145] Gadzalski M, Sakowicz T (2011). Novel SINEs families in *Medicago truncatula* and *Lotus japonicus*: bioinformatic analysis. *Gene*.

